# Caloric restriction increases the resistance of aged heart to myocardial ischemia/reperfusion injury via modulating AMPK–SIRT_1_–PGC_1a_ energy metabolism pathway

**DOI:** 10.1038/s41598-023-27611-6

**Published:** 2023-02-04

**Authors:** Zhijia Guo, Meng Wang, Xiaodong Ying, Jiyu Yuan, Chenggang Wang, Wenjie Zhang, Shouyuan Tian, Xiaoyan Yan

**Affiliations:** 1 1st Hospital of Shanxi Medical University, Shanxi Medical University, Taiyuan, Shanxi China; 2 School of Public Health, Shanxi Medical University, Taiyuan, Shanxi China; 3 Shanxi Traditional Chinese Medicine Hospital, Taiyuan, Shanxi China

**Keywords:** Molecular biology, Zoology, Cardiology, Diseases, Medical research, Pathogenesis

## Abstract

A large number of data suggest that caloric restriction (CR) has a protective effect on myocardial ischemia/reperfusion injury (I/R) in the elderly. However, the mechanism is still unclear. In this study, we created the I/R model in vivo by ligating the mice left coronary artery for 45 min followed by reperfusion. C57BL/6J wild-type mice were randomly divided into a young group fed ad libitum (y-AL), aged fed ad libitum (a-AL) and aged calorie restriction group (a-CR, 70% diet restriction), and fed for 6 weeks. The area of myocardial infarction was measured by Evan’s blue-TTC staining, plasma cholesterol content quantified by ELISA, fatty acids and glucose measured by Langendorff working system, as well as protein expression of AMPK/SIRT_1_/PGC_1a_ signaling pathway related factors in myocardial tissue detected by immunoblotting. Our results showed that CR significantly reduced infarct size in elderly mice after I/R injury, promoted glycolysis regardless of I/R injury, and restored myocardial glucose uptake in elderly mice. Compared with a-AL group, CR significantly promoted the expression of p-AMPK, SIRT_1_, p-PGC_1a_, and SOD_2_, but decreased PPARγ expression in aged mice. In conclusion, our results suggest that CR protects elderly mice from I/R injury by altering myocardial substrate energy metabolism via the AMPK/SIRT_1_/PGC_1a_ pathway.

## Introduction

Ischemic heart disease is the leading cause of mortality worldwide. Coronary intervention is currently one of the most effective methods of restoring blood flow to the ischemic myocardium^1,2^. However, reperfusion itself can cause cardiomyocyte death and increase infarction area, resulting in myocardial ischemia/reperfusion (I/R) damage; this can negatively affect the recovery and prognosis of patients. Age is a risk factor for myocardial I/R injury, and studies have shown that the hearts of older animals are more susceptible to ischemia than those of younger animals^3,4^, and the capacity of myocardium to tolerate ischemia is significantly reduced in aged mice^5,6^. Cardiac I/R susceptibility is strictly regulated by the metabolism of energy substrates^7^. Altered energy substrate utilization, particularly glucose and fatty acid metabolism, is thought to be a key factor in myocardial susceptibility to I/R injury^8,9^. Therefore, regulating the energy metabolism of myocardial substrates may represent an effective approach of protecting against I/R damage.

Caloric restriction (CR) is scientifically proven to promote health and prolong lifespan^10,11^. CR involves a sustained reduction in the intake of pre-intervention energy requirements while maintaining an adequate nutritional supply to achieve weight stability^12^. In experimental animals, CR has been extensively studied as an intervention that can prevent and reverse age-related changes^13–15^. CR exerts cardioprotective effects by antagonizing deleterious phenomena associated with ageing^16–18^. We have previously shown that CR is associated with changes in energy metabolism in organisms, and it reduces stem cell damage by reducing energy intake^19^. CR can also alter cardiac energy metabolism and improve post-ischemic recovery by promoting glucose oxidation and activating the RISK pathway^20^. On the basis of these evidence, we hypothesized that the myocardium energy utilization was altered by CR, which confers protection of aged heart from IR injury in.

CR can activate cardiovascular protection signals^21–23^, such as AMP-activated protein kinase (AMPK) and silencing information regulator 2-related enzyme 1 (sirtuin1, SIRT_1_). AMPK is an important regulator of cellular metabolism and a key regulator of energy homeostasis. Activation of AMPK during I/R exerts cardioprotective effects through regulating multiple metabolic pathways, including glucose and fatty acid metabolism, mitochondrial function, oxidative stress, autophagy, and apoptosis^24–26^. SIRT_1_ is an NAD^+^-dependent deacetylase involved in the regulation of cellular senescence, energy homeostasis, and oxidative stress^27^. SIRT_1_ overexpression protects cardiomyocytes from I/R-induced oxidative damage and cell death^27,28^. Recent studies have shown that AMPK activates SIRT_1_ to regulate the activity of peroxisome-proliferator-activated receptor γ coactivator 1 α (PGC_1a_)^29–31^. For example, resveratrol activates AMPK, increases SIRT_1_ and PGC_1a_ protein levels, and improves muscle mitochondrial respiration on fatty acid derived substrates^29^. Tilianin preconditioning improves mitochondrial energy metabolism and oxidative stress through AMPK/SIRT_1_/PGC_1a_ signaling pathway after I/R injury^31^. However, whether the activation of AMPK/SIRT_1_/PGC_1a_ signaling pathway is, at least partially, responsible for CR beneficial effects in I/R remains elusive.

Therefore, in this study, using mice myocardial I/R model, we investigated the mechanisms of CR protecting the myocardium from I/R injury by measuring the changes of myocardial energy metabolism substrates and metabolic signals of substrates after CR intervention.

## Result

### CR decreases mice body weight

**Figure 1:**
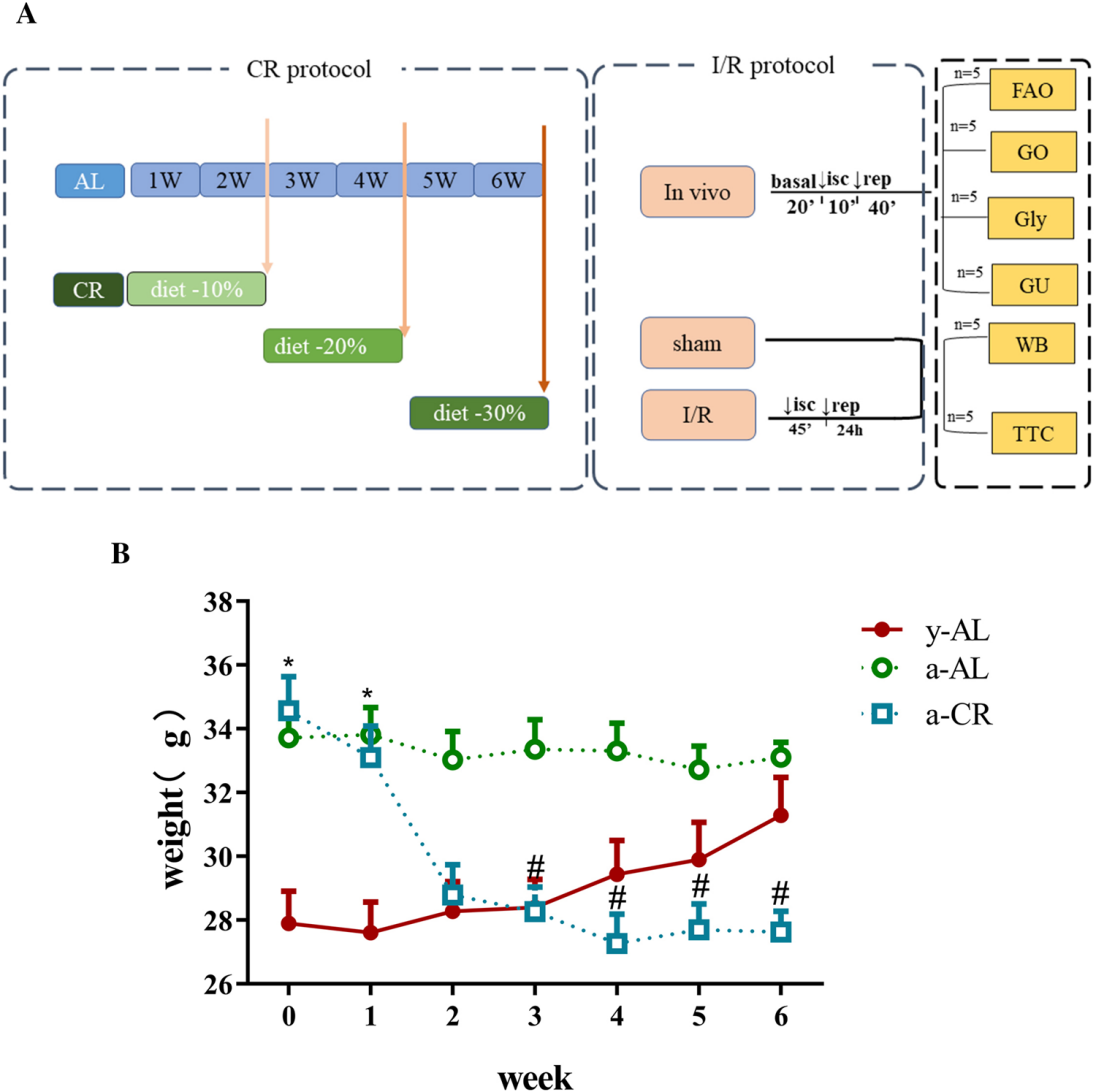
CR decreases mice body weight. (**A**) Experimental design. A 6-week model of caloric restriction CR and a myocardial I/R injury model were established. *AL* ad libitum, *CR* caloric restriction, *FAO* fatty acid oxidation, *GO* glucose oxidation, *Gly* glycolysis, *GU* glucose uptake. (**B**) Body weight changes of mice in each group during 6-week caloric restriction. *P < 0.05 vs y-AL; ^#^*P* < 0.05 vs a-AL.

The experimental protocols for CR and I/R injury were depicted in Fig. 1A. Animal body weight was recorded weekly for 6 weeks. As shown in Fig. 1B, during the 6-week ad libitum period, the y-AL mice gradually gained weight, but the a-AL animals did not show any significant changes in body weight. In contrast, the a-CR mice had a significantly higher body weight at weeks 0 and 1 than the y-AL group; there was a dramatic weight loss starting from week 2 in the a-CR animals.

### CR reduces plasma cholesterol levels

**Figure 2:**
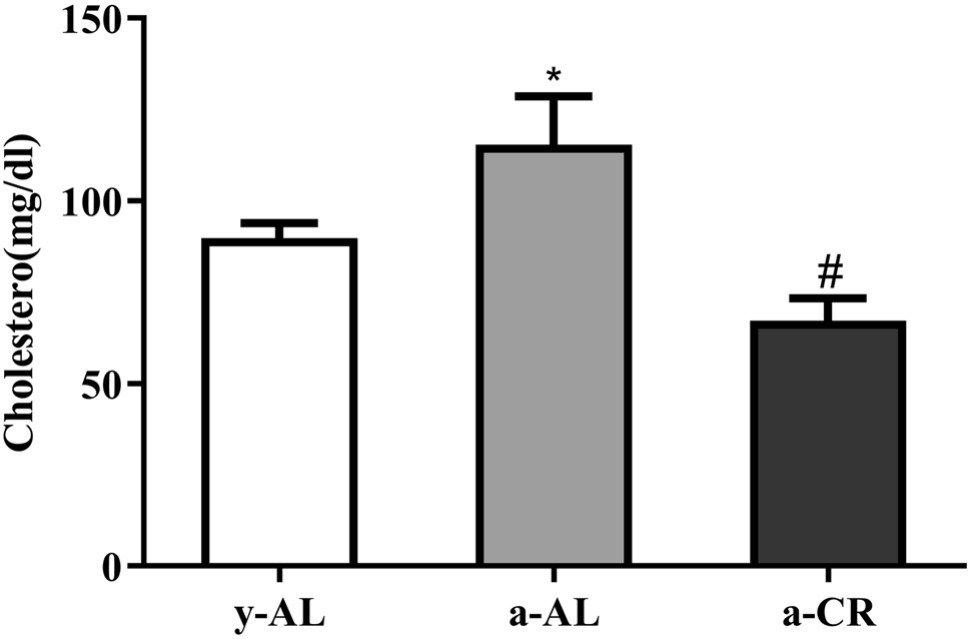
Changes in plasma cholesterol. Plasma cholesterol was detected by ELISA. The values of each group were mean ± SEM, N = 5–10. *P < 0.05 vs y-AL; ^#^*P* < 0.05 vs a-AL.

To determine how CR affects blood lipid levels, we measured plasma cholesterol concentration. As shown in Fig. 2, total plasma cholesterol levels were higher in the a-AL group compared to the y-AL group (*P* < 0.05), while plasma cholesterol was reduced in the a-CR mice compared to the a-AL group (*P* < 0.05), indicating that CR can reduce plasma cholesterol levels.

### CR limits the infarction of myocardium

**Figure 3:**
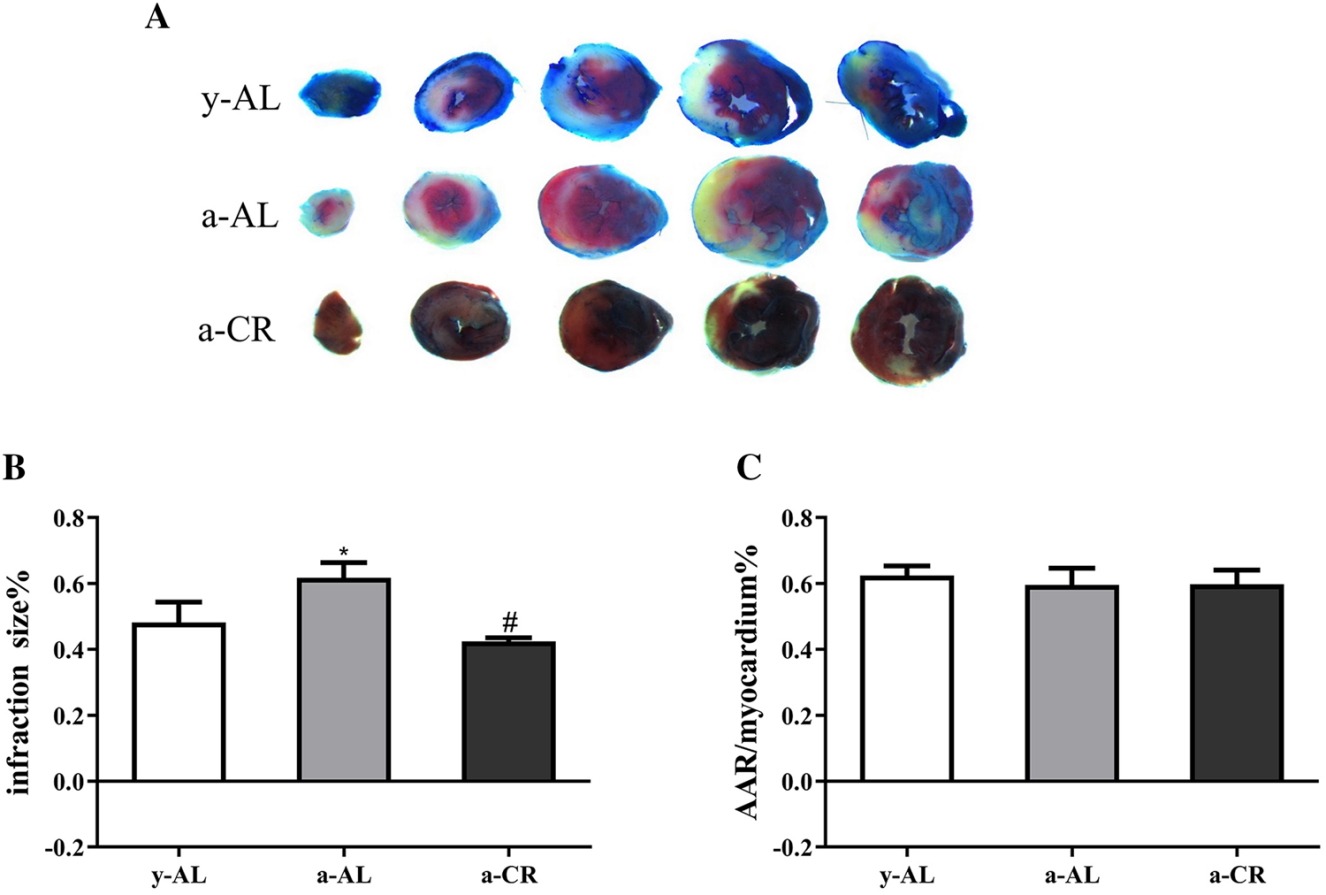
Size of myocardial infarction determined by Evan’s blue and TTC staining. (**A**) The result of Evan’s blue and TTC staining. The blue area is normal, and the remaining areas (including red and white) are ischemic risk zones (AAR), with white areas being myocardial infarction (IA) and red areas being ischemic but not infarcted. (**B**) Infarct size% was the ratio of IA to AAR. (**C**) AAR% was the ratio of AAR to the whole heart slide area. N = 5 for each group, group: *y-AL* youth fed ad libitum group, *a-AL* aged fed ad libitum group, *a-CR* aged caloric restriction group. One-way ANOVA was used to analyze the data. *P < 0.05 vs y-AL; ^#^*P* < 0.05 vs a-AL.

Myocardial infarct area (Fig. 3A) was quantified using Evan’s blue and TTC staining 24 h after reperfusion. We observed that the infarct area was significantly higher in the a-AL group than in the y-AL group (*P* < 0.05), which was significantly reduced in the a-CR group (*P* < 0.05) (Fig. 3B); by contrast, the area at risk (AAR%) was similar in all groups (Fig. 3C). These data indicate that CR decreases infarct size.

### CR induces apoptosis in the peri-infarct of myocardium

**Figure 4:**
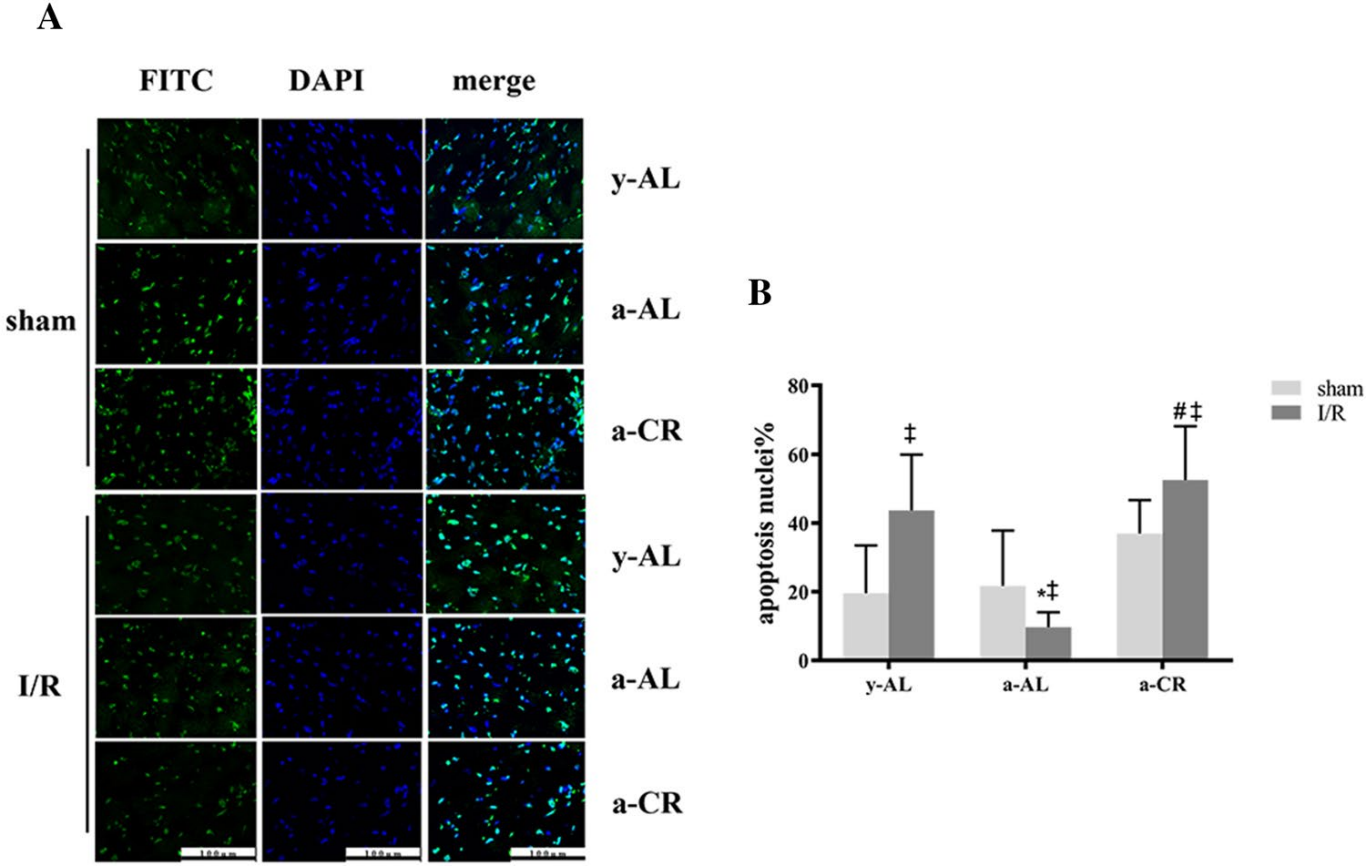
CR induces apoptosis in the peri-infarct myocardium of aged mice. (**A**) Representative images of apoptosis (in green) evaluated with TUNEL staining for. Nuclei were depicted by DAPI in blue. (**B**) Quantification of TUNEL positive (a) apoptotic cardiomyocyte nuclei in frozen sections from peri-infarct region after 24 h reperfusion. Data were from 6 randomly selected images. The group: *y-AL* young ad libitum group, *a-AL* aged ad libitum group, *a-CR* aged caloric restriction group. *P < 0.05 vs y-AL; ^*#*^*P* < 0.05 vs a-AL; ^‡^*P* < 0.05 vs sham operation.

To further assess the protective effect of CR on I/R heart, we used a TUNEL kit to detect myocardial apoptosis (Fig. 4A). The results showed that after I/R injury, the number of apoptotic cells in y-AL group and a-CR group increased significantly. However, the number of apoptotic cells in a-AL group decreased significantly compared with that in sham operation group. In addition, after I/R injury, the number of apoptotic cells in a-CR group was significantly higher than that in a-AL group (Fig. 4B). This suggests that CR can promote cell apoptosis, clear aging and damaged cells, accelerate organ damage repair and maintain body health.

### CR increases glucose metabolites and decreases fatty acid oxidation in aged I/R heart

**Figure 5:**
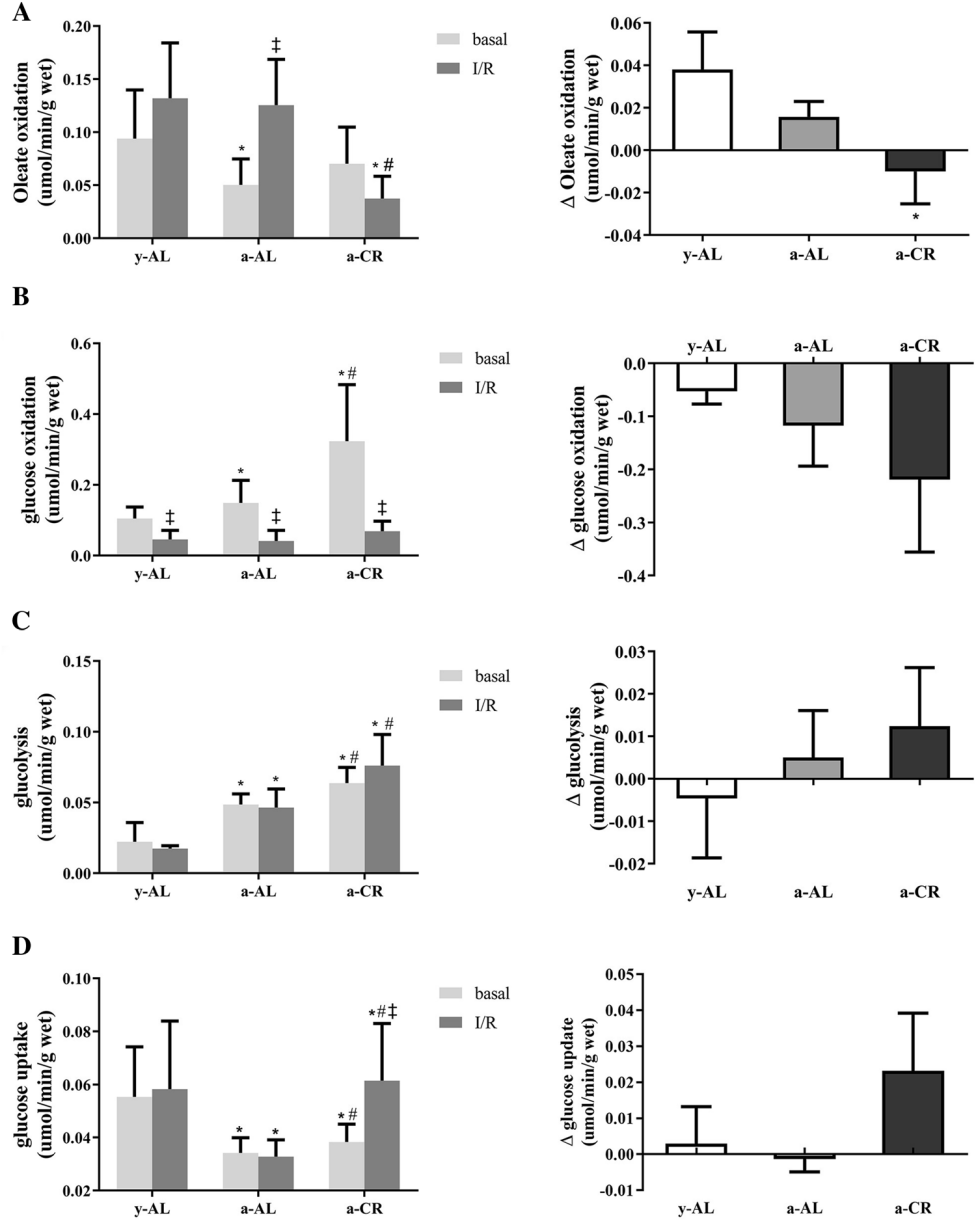
Analysis of substrate metabolism in the hearts. Oleic acid oxidation (**A**) and glucose oxidation (**B**) were measured in isolated working hearts. The hearts were intravenously infused with buffers containing [9, 10-3H_2_O] and [^14^C] for equilibrium for 20 min, then underwent ischemia for 10 min, followed by reperfusion for 40 min. Glycolysis (**C**) and glucose uptake (**D**) were measured using d-[5–3h] glucose or d-[2–3h] glucose in the Langendorff system, respectively. The delta (Δ = rep-basal) gradient between aerobic and reperfusion was calculated in all four graphs. *P < 0.05 vs y-AL; ^#^*P* < 0.05 vs a-AL; ^‡^*P* < 0.05 vs the base value.

The gradient between aerobic and reperfusion was calculated using delta (Δ = rep-basal) baseline (Fig. 5, right column). At the basal state, oleic acid oxidation was significantly lower in the a-AL group than in the y-AL group (*P* < 0.05), and this difference disappeared when aged mice were challenged with CR; after I/R, oleic acid oxidation was elevated in the a-AL group compared with the basal state (*P* < 0.05), whereas it was significantly lower in the a-CR group compared with the other two groups (*P* < 0.05). At the basal state (Fig. 5B), glucose oxidation levels were significantly higher in the a-AL and a-CR groups than in the y-AL group, and also significantly higher in the a-CR than in the a-AL group (*P* < 0.05). During reperfusion, glucose oxidation levels in all groups were in a depressed state, probably inhibited by the oleic acid oxidation response (Fig. 5B). At the basal state, glycolysis levels were higher in the a-AL group than in the y-AL group (*P* < 0.05), which were further elevated by CR (*P* < 0.05) (Fig. 5C). Although glucose uptake in the a-AL group was significantly lower than y-AL group (*P* < 0.05) at the basic state, CR tended to recover glucose uptake in the heart of aged mice (Fig. 5D). All of these data suggested that CR can increase glucose metabolites and reduce fatty acid oxidation in the aged heart against myocardial I/R injury.

### CR activates the AMPK/SIRT_1_/PGC_1a_ signaling pathway

**Figure 6:**
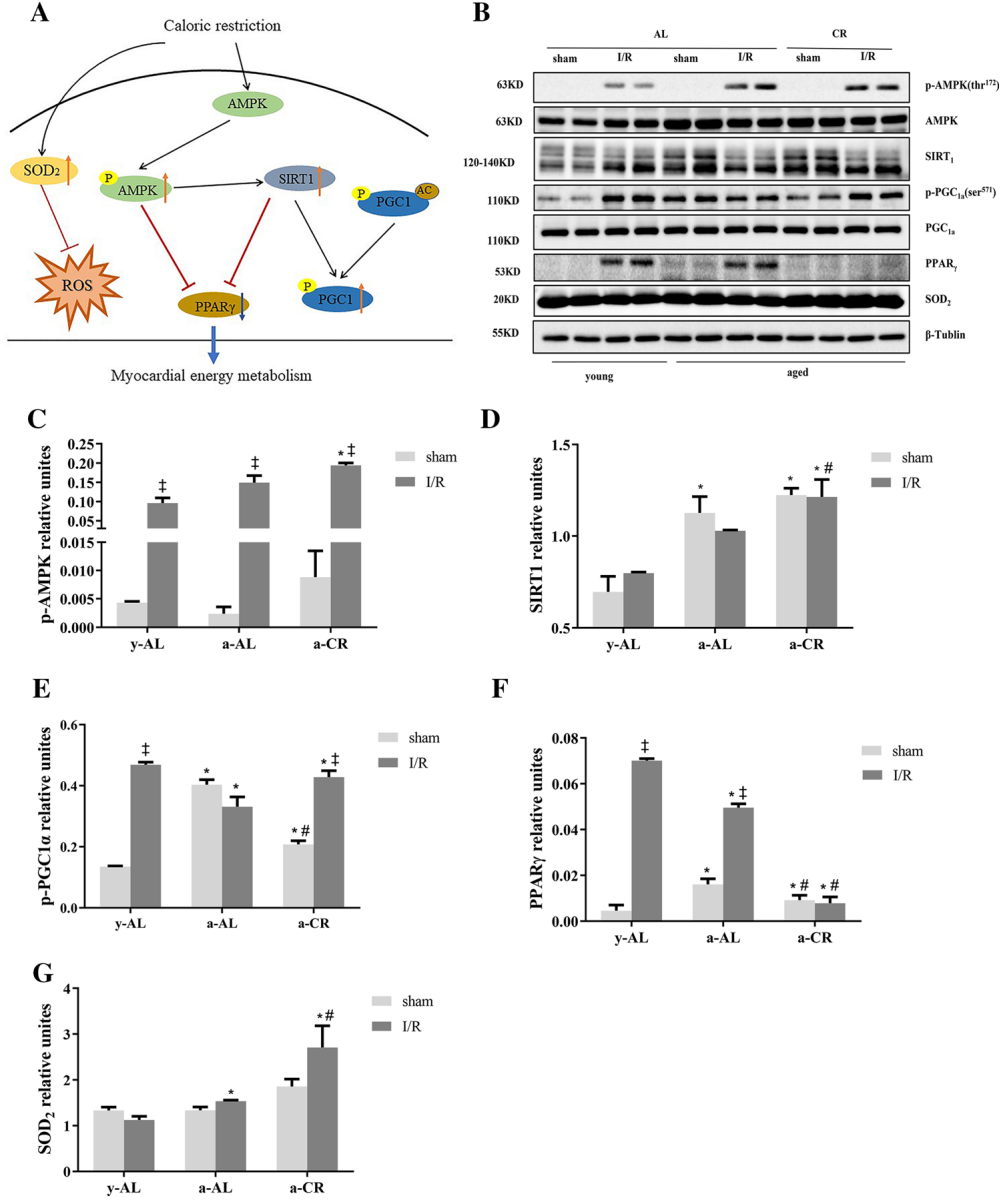
Expression of cardiometabolic proteins. (**A**) AMPK/SIRT_1_/PGC_1a_ signaling pathway. CR leads to activation of AMPK. AMPK regulates SIRT_1_ by inducing NAD^+^. SIRT_1_ activates PGC_1a_ by phosphorylation and deacetylation. AMPK and SIRT_1_ synergistically inhibit PPARγ. CR increases SOD_2_ expression in vivo and inhibits ROS. (**B**) Representative images of immunoblotting for p-AMPK, SIRT_1_, p-PGC_1a_, PPAR-γ, and SOD_2_. (**C**) Quantitative results of p-AMPK protein. (**D**) Quantitative results of SIRT_1_ protein. (**E**) Quantitative results of p-PGC_1a_ protein. (**F**) Quantitative results of PPAR-γ protein. (**G**) Quantitative results of SOD_2_ protein. The westerns were cut prior to incubation with the primary antibodies, the full size blots can be seen in Supplementary Fig. S1. Western blotting showed protein abundance in each group, N = 5, *y-AL group* youth fed ad libitum group, *a-AL* aged fed ad libitum group, *a-CR* aged caloric restriction group. *P < 0.05 vs y-AL; ^#^*P* < 0.05 vs a-AL; ^‡^*P* < 0.05 vs sham operation.

To explore how CR alters substrate metabolism in the heart, we measured the expression of important signaling molecules for energy metabolism in heart tissues (Fig. 6A,B). AMPK is not only an important regulator of energy metabolism but also a stress signal under I/R injury in the heart. Phosphorylated AMPK was significantly higher in a-CR hearts than y-AL, both under basal and I/R conditions (P < 0.05) (Fig. 6C). AMPK regulates SIRT_1_ by inducing NAD^+^^30^. In both sham and I/R groups, there was a notable higher level of SIRT_1_ in CR group than young group (P < 0.05) (Fig. 6D). AMPK and SIRT_1_ activate PGC_1a_ by phosphorylation^30^. P-PGC_1a_ was downregulated in a-AL group but upregulated in y-AL and a-CR after I/R (Fig. 6E). In general, the PPARγ coordinates adipogenesis and glucose homeostasis, and is inhibited by SIRT_1_. We found that PPARγ was significantly blunted in aged I/R heart by CR (P < 0.05) (Fig. 6F). Additionally, there was a significant upregulation of antioxidant SOD_2_ in a-CR group after I/R, which was not seen in y-AL and a-AL mice (P < 0.05) (Fig. 6G). Overall, CR activates the AMPK/SIRT_1_/PGC_1a_ signaling pathway to protect the aged heart from I/R injury.

## Discussion

I/R injury results in myocardial energy metabolism dysfunction and ATP deficiency, which causes a variety of injuries, including cationic pump dysfunction, calcium overload, and overproduction of reactive oxygen species^29^. Therefore, restoring energy metabolism in cardiomyocytes is considered as a potential strategy to alleviate myocardial I/R injury. CR is a nutritional intervention that reduces energy intake while providing sufficient nutrition^32^, which can affect energy metabolism and mitochondrial function in the body. Metabolic changes induced by aging occur at different levels; and we wanted to determine whether CR affects cardiomyocyte metabolism in the aged mice to improve recovery following I/R.

There is growing evidence that CR has a protective effect against the development and progression of cardiovascular disease^33,34^ Long-term CR can reduce weight and lower blood pressure, fasting plasma glucose, insulin, and cholesterol levels. In addition, CR can keep older animal physiological states “young”, which was consistent with our results showing that a-CR mice exhibit reduced body weight and lower total plasma cholesterol levels compared to the a-AL group. Intermittent fasting initiated before or after myocardial infarction reduces cardiomyocyte hypertrophy and left ventricular dilation^35^. Reducing caloric intake reduces myocardial infarct and improves myocardial function after myocardial infarction^36^. In line with above findings, our study found that older mice had larger infarct size after acute I/R than young mice, and CR inhibited infarct size in old mice. Interestingly, CR increased apoptosis in the peri-infarct myocardium. Apoptosis can promote the clearance of senescent or damaged cells and accelerate tissue repair^37,38^. How CR inhibited ischemia-induced necrosis to limit infarct size but promoted apoptosis needs to be investigated in future studies.

CR improves substrate energy metabolism after cardiac I/R injury. In the normal aerobic heart, β-oxidation of fatty acids is the main source of myocardial energy, accounting for approximately 60% of total energy, with the remainder coming mainly from carbohydrates, particularly glucose. ATP production from both fatty acid oxidation and glucose oxidation is dependent on the presence of oxygen; in contrast, glycolysis occurs under anaerobic conditions. During ischemia, oxygen supply is restricted, fatty acid and glucose oxidation is reduced, but anaerobic glycolysis is increased^39,40^. However, during reperfusion fatty acid oxidation is rapidly restored and glucose oxidation is inhibited. Interestingly, glycolysis is unaffected by these high rates of fatty acid oxidation and remains elevated during I/R^41^. Enhanced fatty acid oxidative metabolism during I/R has been reported to exacerbate I/R injury^7,42^. Our results revealed enhanced fatty acid metabolism and attenuated glucose oxidation in y-AL and a-AL hearts after the occurrence of I/R, whereas the elderly CR intervention mice had increased glucose uptake and reduced fatty acid oxidation, suggesting that CR intervention optimized myocardial energy metabolism in the elderly and alleviated the adverse consequences of I/R.

CR activates the expression of AMPK/SIRT_1_/PGC_1a_ signaling pathway in response to myocardial energy changes. AMPK, as a regulator, is a key player in ischemia-induced alterations of fatty acid and glucose metabolism. Accumulating evidence suggests that AMPK activation improves energy metabolism during ischemia, leading to rapid changes in fatty acids and glucose oxidation by increasing glucose uptake, glycolysis rate, and ATP production^43^. Tilianin^31^ preconditioning can improve mitochondrial energy metabolism and oxidative stress through AMPK/SIRT_1_/PGC_1a_ signaling pathway in rats after myocardial I/R injury. Our data demonstrated that CR intervention promoted the protein expressions of p-AMPK, AMPK, SIRT_1_ and PGC_1a_, which may activate AMPK/SIRT_1_/PGC_1a_ signaling pathway to alleviate myocardial injury in the elderly. Peroxisome proliferators-activated receptor γ (PPARγ) can mediate adipogenesis and glucose homeostasis, but p-AMPK and SIRT_1_ can synergically inhibit their expression and prevent lipid synthesis^44^. Superoxide dismutase 2 (SOD_2_) is known to have strong resistance to ROS oxidative stress and inflammatory cytokines. We found that CR attenuated PPAR γ and upregulated SOD_2_ in aging I/R hearts. In conclusion, CR not only optimizes the energy metabolism of myocardial substrates, but also reduces the damage caused by oxidative stress after I/R.

Although our data clearly suggest the beneficial effect of optimizing energy metabolism by CR in the aged heart, more rigorous work delineating the effect is warranted. Since the change in metabolism by CR is so profound, investigating the function of cardiomyocyte mitochondria is an important research point. This study only observed the situation within 24 h after reperfusion, and longer time points are needed. In addition, using genetically knockout animals can convincingly verify metabolic changes induced by CR.

## Conclusion

In the present study we found that CR significantly inhibited I/R-induced myocardial injury in aged mice, as evidenced by a reduction in myocardial infarct size. In addition, CR improved myocardial energy impairment induced by I/R by promoting glycolysis levels and restoring myocardial glucose uptake in aged mice. Mechanistically, we provided the first evidence that CR improved myocardial energy metabolism after I/R in aged mice by activating the AMPK/SIRT_1_/PGC_1a_ signaling pathway and promoted SOD_2_ expression to resist oxidative stress injury. These findings provide a theoretical basis for future investigations into the mitigating effects of CR on I/R-induced myocardial injury.

## Materials and methods

### Experimental animals

Sixty young (3 months old) and aged (18 months old) C57BL/6J male mice were selected and purchased from Charles River Laboratory, USA. The temperature of the animal room was controlled at 20–25 °C, and the lighting was alternated between day and night at 12 h/12 h. Procedures were approved by the Institutional Animal Care and Use Committee at the University of Mississippi Medical Center and were conducted in accordance with the Guide for the Care and Use of Laboratory Animals.

### Experimental materials

2% Isoflurane, 8-0 polyprolene suture, buprenorphine 0.01 mg/kg, 2% Evan’s Blue, 1% 2, 3, 5-triphenyltetrazole (TTC, Sigma, T8877-100g), Leica microscope (Germany), biochemical analyzer, d-glucose 7 mM, insulin 10 mU/L, Oleate 0.4 mM, 1% bovine serum albumin (BSA), [9, 10]-^3^H-oleic acid (50 mci/L), ^14^C-glucose (20 mci/L), d-[2–3H] glucose (1 mci/L) and d-[5–3H] glucose (1 mci/L) were purchased from Perkin Elmer Company in the United States.

### Animal grouping and CR model establishment

Young (y) and aged (a) C57BL/6J male mice were randomly divided into three groups: young fed ad libitum (y-AL) group, aged fed ad libitum (a-AL) group, and aged 70% caloric restriction (a-CR) group. In CR groups, animals were housed in individual cage, and the initial baseline was the average of daily food intake during the first AL week (week 0). Calorie restriction begins after the first AL week: initial food intake (i.e., average food intake in the first AL week) was reduced by 10% every 2 weeks and total food intake was reduced by 30% by 6 weeks, with body weight loss < 20%. The group of y-AL and a-AL mice have free access to standard chow and drinking water. The weekly changes in body weight of mice were observed and recorded. After 6-week CR, the animals were subjected to I/R injury or sham surgery.

### Myocardial I/R injury model

Animals were anaesthetized with isoflurane (2%), and body temperature was kept at 37 °C. A left thoracotomy was performed, and the ribs were spread apart to expose the left ventricle (LV). Ischemia was induced by ligating the left anterior descending (LAD) branch of coronary artery with an 8–0 polyprolene suture. An electrocardiogram (ST-segment elevation) and blanching of LV confirmed cardiac ischemia. After 45 min of ischemia, the suture was released to induce reperfusion. Once the skin was sutured back together, an intraperitoneal injection of buprenorphine (0.01 mg/kg) was provided for analgesia. The animals were allowed to recover for 24 h after reperfusion. The sham-operated mice underwent the same procedure with the exception of artery ligation.

### Determination of myocardial infarction area

Twenty-four hours after perfusion, the LAD was ligated, and 0.3 mL of 2% Evan’s blue was injected into the internal jugular vein. The heart was taken out immediately after injection, rinsed with PBS and sectioned. The slices were then immersed in TTC phosphate buffer solution for 5 min at 37 °C. Ischemic areas were stained blue by Evan’s blue and normal myocardium is stained red by TTC. The stained heart sections were photographed with a Leica microscope, and the infarct size was analyzed with ImageJ software.

### TUNEL staining

The heart was quickly removed and cut into halves from the center of left ventricle along the long axis, one for frozen sections. Fresh heart tissue was placed in optimal cutting temperature (OCT) compound embedding agent, frozen at − 20 °C, cut into 5 μm slices. Slices were fixed in 4% paraformaldehyde, permeabilized with 0.1% Triton X-100 for 2 min. Apoptosis assay was carried out according to the instructions of TUNEL kit (Roche, US) and images were analyzed using a fluorescence microscopy.

### Measurement of plasma biochemical indexes

Mice blood was collected from the eyes after successful anesthesia with ether. The blood was immediately placed in centrifuge tubes pre-treated with sodium heparin and centrifuged at 4 °C, 4000 rpm for 15 min to collect the plasma. Plasma cholesterol content was determined by ELISA kit.

### Measurement of oleate and glucose oxidation, glycolysis, glucose uptake

Oleate and glucose oxidation, glycolysis, and glucose uptake were tested with a working heart and Langendorff system. Briefly, under anesthesia, the hearts were removed quickly and perfused with modified Krebs–Henseleitbuffer containing d-glucose (7 mM), insulin (10 mU/L), oleate (0.4 mM), BSA (1%) according to the langendorff procedure. The working heart system keeps the pre-load at 15 cmH_2_O, the after-load at 80 cmH_2_O, and flow rate at 15 mL/min. [9, 10]-^3^H-oleate (50 mci/L, PerkinElmer, US) and ^14^C-glucose (20 mci/L, PerkinElmer, US) labeled BSA buffer were perfused into the pulmonary vein and pumped out of the aorta. Perfusate was recycled and collected every 5 min to test the radioactivity, and ^3^H and ^14^C signals were detected to discriminate metabolic products from fatty acid and glucose, respectively. The fatty acid level was determined by the production of [9, 10]-^3^H_2_O from oleate by filtering through anion-exchange 1-X2 resin (Bio-Rad, Hercules, CA, US). Meanwhile, glucose oxidation was measured by both the ^14^CO_2_ gaseous dissolved in sodium hydroxide and ^14^CO_2_ separated from ^14^C-glucose by sulfuric acid.

For glucose uptake and glycolysis measurements, perfused radio buffer was recycled only through aorta and collected every 5 min to test the radioactivity. Metabolized ^3^H_2_O was separated from d-[2–3H] glucose (1 mci/L, PerkinElmer, US) or d-[5–3H] glucose (1 mci/L, PerkinElmer, US) by filtering through anion-exchange 1-X8 resin (Bio-Rad, Hercules, CA, USA). The rate of glucose uptake and glycolysis was calculated by the amount of ^3^H_2_O production. Scintillation fluid (10 mL) was added to each vial and mixed well to measure radioactive signal on a liquid scintillation counter.

### Western blot

Cardiac tissue (30 µg) from the same part of the heart was homogenized with RIPA lysate containing protease and phosphatase inhibitors. After centrifugation at 12,000 r/min for 15 min at 4 °C, the supernatant was collected. Protein concentration was determined using a BCA protein assay kit (Beyotime Institute of Biotechnology, China). Samples were separated by 8% SDS-PVDF gel electrophoresis and transferred to PVDF membranes. The samples were incubated in 5% BSA in the blocking solution for 2 h at room temperature, incubated overnight at 4 °C with corresponding primary antibodies, washed with Tris-Buffered Saline Tween-20 (TBST) 3 times, incubated for 1 h at room temperature with secondary antibodies. Rabbit antibodies P-AMPKα (thr172,2535), AMPKα (5831s), PPAPγ (2443s), and SOD_2_ (13141), were purchased from Cell Signaling Technology (UK) rabbit anti-P-PGC_1a_ (ser571, AF6650), and PGC_1a_ (NFP1-04676) from Novus Biologics, and SIRT_1_ monoclonal antibody (ab12193) from Abcam (USA). The target signals were detected using ECL luminescence reagents and an electrophoretic gel imaging and analysis system.

### Statistical analyses

Statistical analysis was performed using SPSS 22.0 software (IBM, Chicago, IL, USA). All values are presented as mean ± SE (n observations). The significance of differences was determined by the use of a two-tailed Student's *t* test and analysis of variance (ANOVA). Significant differences are denoted *P* < 0.05.

### ARRIVE guidelines

The study was conducted in compliance with the ARRIVE guidelines and all methods were conducted in accordance with relevant guidelines and regulations.

### Ethical approval

The investigation conforms to the Guide for the Care and Use of Laboratory Animals published by the US National Institutes of Health (NIH Publication No. 85-23, revised 1996). The University of Mississippi adheres to the principles for biomedical research involving animals developed by the Council for International Organizations of Medical Sciences and complies with the US Council on Animal Care guidelines. All animal procedures were approved by the UMMC Health Sciences Animal Welfare Committee.

## Supplementary Information

Supplementary Figure S1.

## Data Availability

The datasets used and analyzed during the current study are available from the corresponding author on reasonable request.
