# Supplementary material for: Caloric restriction increases the resistance of aged heart to myocardial ischemia/reperfusion injury via modulating AMPK–SIRT1–PGC1a energy metabolism pathway
Source: Sci Rep. 2023 Feb 4;13:2045. doi: 10.1038/s41598-023-27611-6 (PMC9899227; doi:10.1038/s41598-023-27611-6)

Supplementary Information

Journal: Scientific Reports

Manuscript ID: 1f386712-1784-4ab4-a469-de219d04a7ab

Title: Caloric restriction increases the resistance of aged heart to myocardial ischemia/reperfusion injury via modulating AMPK-$SIRT_1$-PGC_{1a} energy metabolism pathway

Author(s): Zhijia Guo*, Meng Wang, Xiaodong Ying, Jiyu Yuan, Chenggang Wang, Wenjie Zhang, Shouyuan Tian, Xiaoyan Yan*,

Co-correspondence Author(s): Zhijia Guo, Xiaoyan Yan

Supplementary Fig. S1 - Uncut westernblots from Fig. 6 Blots were cut before antibody incubatio

A.P-AMPK

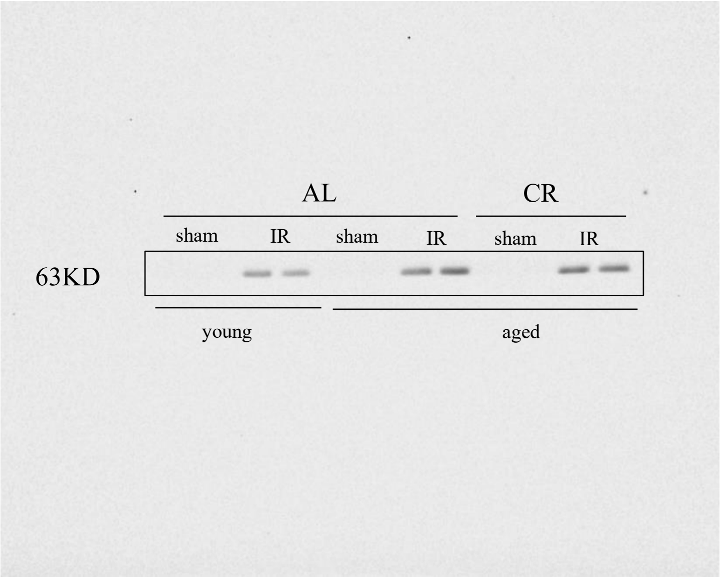

AMPK

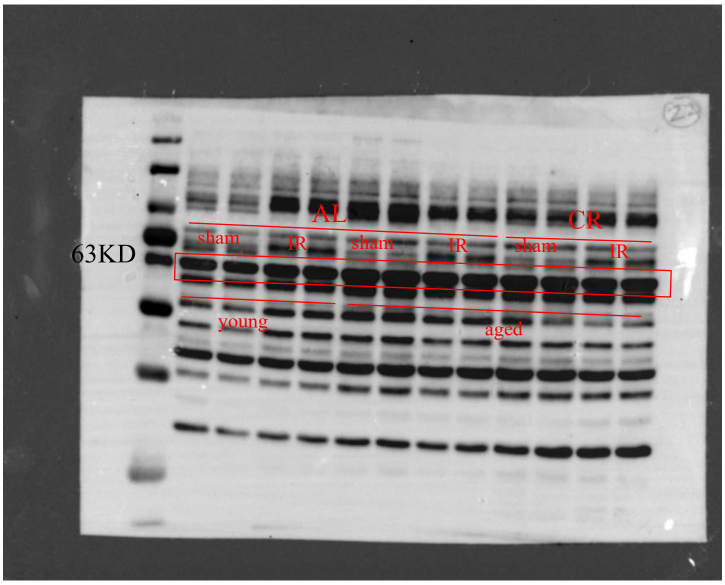

B. SIRT1

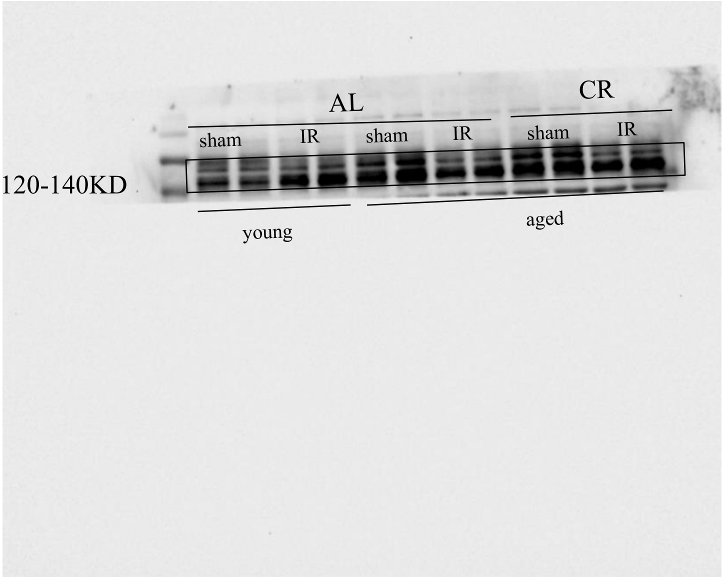

C.p-PGC1a

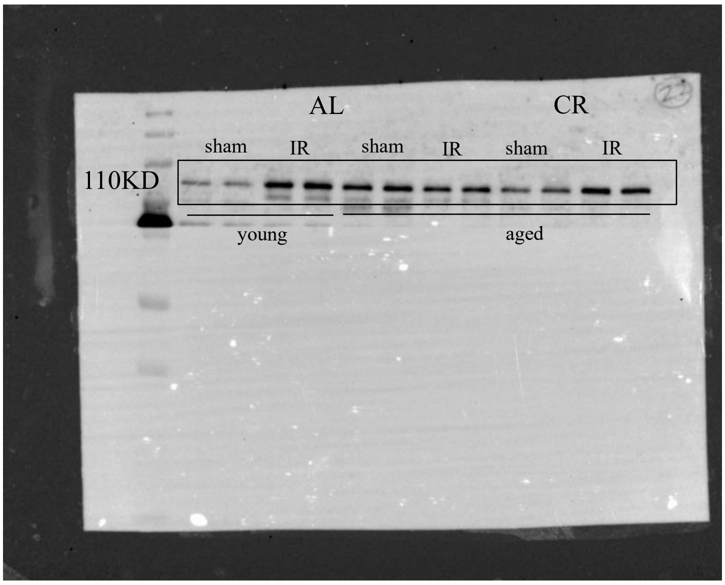

PGC1a

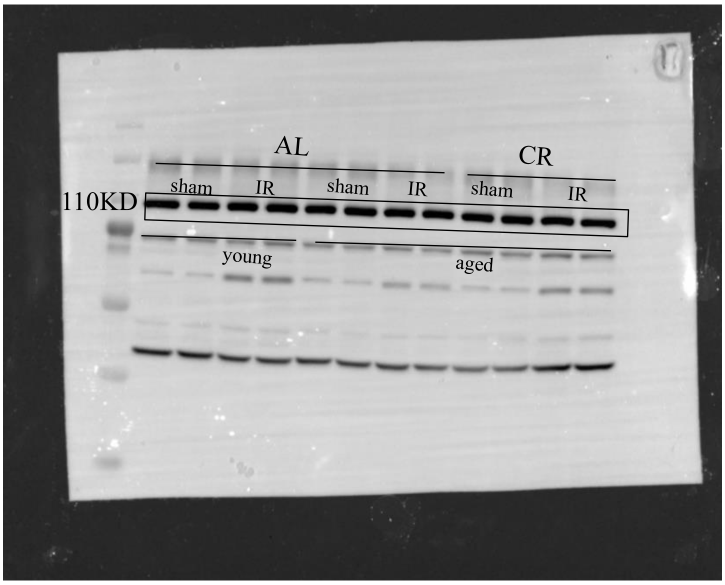

D. PPARγ

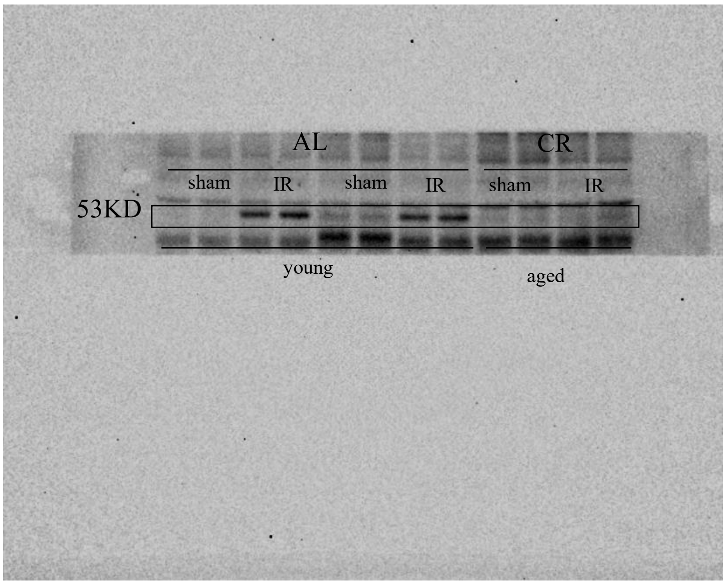

E. SOD2

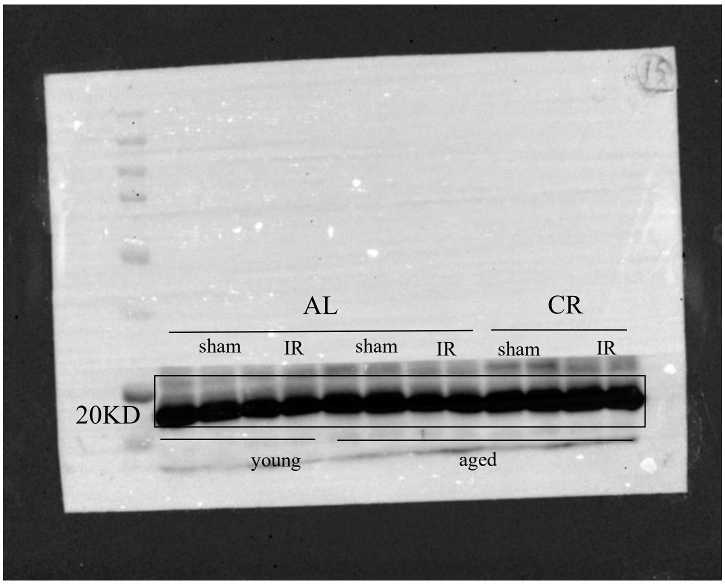

F. β-Tublin

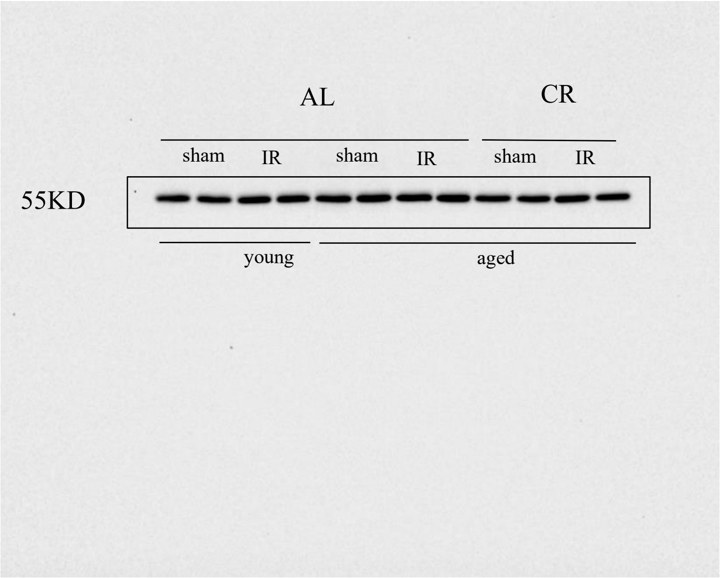

Supplement: Supplementary file 1 — Supplementary Figure S1. [file 41598_2023_27611_MOESM1_ESM.pdf]
